# N6-Methyladenosine Modification Participates in the Progression of Hepatitis B Virus-Related Liver Fibrosis by Regulating Immune Cell Infiltration

**DOI:** 10.3389/fmed.2022.821710

**Published:** 2022-03-02

**Authors:** Tong Zhao, Jianni Qi, Tiantian Liu, Hao Wu, Qiang Zhu

**Affiliations:** ^1^Department of Gastroenterology, Shandong Provincial Hospital, Cheeloo College of Medicine, Shandong University, Jinan, China; ^2^Central Laboratory, Shandong Provincial Hospital Affiliated to Shandong First Medical University, Jinan, China; ^3^Department of Infectious Diseases, Shandong Provincial Hospital Affiliated to Shandong First Medical University, Jinan, China; ^4^Department of Infectious Diseases, Shandong Provincial Hospital, Cheeloo College of Medicine, Shandong University, Jinan, China; ^5^Department of Gastroenterology, Shandong Provincial Hospital Affiliated to Shandong First Medical University, Jinan, China

**Keywords:** N6-methyladenosine, hepatitis B virus, liver fibrosis, immune cell infiltration, immunity

## Abstract

**Aim:**

N6-methyladenosine (m6A) modification has been demonstrated to play an important part in hepatitis B virus (HBV) infection and immune response. This study aims to further investigate whether m6A modification plays an important role in the progression of HBV-related liver fibrosis through the regulation of immune cell infiltration.

**Methods:**

In this study, 124 chronically HBV infected cases were obtained from the Gene Expression Omnibus database. In total, 489 m6A-and-stage related genes were selected to be associated with the m6A modification and the stage of liver fibrosis. Based on these genes, we identified two distinct gene clusters, gene clusterA and gene clusterB. The immune characteristics of the two clusters were comprehensively compared. The m6A-S score was constructed as quantification of individual m6A status. The correlations between m6A regulators and infiltrating immune cells were examined and compared in different pairs of groups with various m6A traits.

**Results:**

Biological functions, immune cell infiltration, and cytokines expression were compared between the two gene clusters proving that the gene clusterB was more immune active and had a more advanced liver fibrosis stage. The m6A-S score was associated with immune infiltration and the progression of liver fibrosis. Five different grouping conditions with different m6A traits were set up. According to the intersection of significant genes and cells, *ALKBH5* interacting with macrophage and *WTAP* interacting with nature killer T cells may be key points in the progress of liver fibrosis.

**Conclusions:**

N6-methyladenosine modification is closely related to the immune cell infiltration and the fibrosis stage of chronic HBV-infected liver tissue. It provides us a better understanding of the progression of liver cirrhosis *via* evaluating the m6A modification pattern and immune infiltration characteristics.

## Introduction

Almost one-third of the human population has been infected by HBV at some point in their lives according to the WHO data (1). Despite the common application of vaccines and antiviral drugs, more than 250 million individuals worldwide developed a persistent infection called chronic hepatitis B (CHB), which can advance to hepatic fibrosis, cirrhosis, and hepatocellular carcinoma (HCC) leading to nearly 1 million deaths annually (1–3). The progress of HBV-related liver fibrosis can be measured by liver biopsy and several scoring systems have been developed to assess this (4). The Scheuer's staging score (Scheuer score “S”) is one of the most commonly acknowledged and applied whose histological evaluation is based on the extent of fibrosis and development of cirrhosis (4–6). However, mechanisms responsible for the progression of liver fibrosis in patients with CHB are incompletely understood.

More than 150 RNA modifications have been found in eukaryotes, such as 5-methylcytosine, N1-methyladenosine, uridine to pseudouridine, and N6-methyladenosine (m6A) (7). Among them, m6A, the most abundant and prominent modification of cellular RNAs, is a dynamic and reversible process regulated by methyltransferases (“writers”), demethylases (“erasers”), and binding proteins (“readers”) (8, 9). The methylation of the sixth position N of adenine on mRNA is catalyzed by the methyltransferase complex composed of *METTL3, METTL14*, and *WTAP* assisted by other functionally related molecules (10, 11), while the reverse process is mediated by demethylases, *FTO*, and *ALKBH5* (12). The dynamic balance of the sites of m6A modification can be recognized by RNA binding proteins, which affects the subsequent biological processes (12, 13). Nowadays, m6A modification has been confirmed to play essential roles in numerous biological responses, such as immune responses, metabolic disorders, viral infection, and cancer development (13–23). However, it remains uncertain whether m6A modifications mediated by these regulators take part in the progress of HBV-related liver fibrosis.

In our study, we identified two distinct m6A-and-stage related gene clusters that proved to have different stages of fibrosis and immune activities. Then, we compared the correlations between m6A regulators and infiltrating immune cells in 5 different pairs of m6A modification groups, through which, critical genes and immune cells were selected.

## Materials and Methods

### Dataset Acquisition and Preprocessing

Contributed by Wang's team, the GSE84044 dataset containing 124 chronically HBV infected patients was obtained from the Gene Expression Omnibus database. According to their report, these patients were hospitalized in Shanghai Ruijin Hospital, Shanghai Huashan Hospital, Shanghai Public Health Clinical Center, and Shanghai General Hospital between 2009 and 2011. They had excluded individuals with concurrent hepatitis C virus, hepatitis D virus or HIV infection, autoimmune liver disease, drug-induced liver injury, alcoholic liver disease, or hepatocellular carcinoma and all patients had not taken any antiviral therapies or immunosuppressive drugs within 6 months before sampling. Public gene-expression data and clinical annotation were downloaded and retrospectively analyzed. Among them, pathological Scheuer scores representing the stage of disease progression (Scheuer S) had been measured and confirmed by two experienced pathologists independently. GSE84044 contains expression profiles of 43 samples in S0, 20 samples in S1, 18 samples in S2, 33 samples in S3, and 10 samples in S4. Their experiments were conducted on the Affymetrix HG U133 Plus 2.0 microarray (GPL570 platform, Affymetrix, Cleveland, Ohio, USA) (24).

In total, 28 widely recognized m6A RNA methylation regulators were collated from published literature. These regulators consisted of 10 writers (*METTL3, METTL14, WTAP, KIAA1429, RBM15, RBM15B, ZC3H13, ZNF217, METTL4*, and *CBLL1*), two erasers (*FTO* and *ALKBH5*), and 16 readers (*YTHDC1, YTHDC2, YTHDF1, YTHDF2, YTHDF3, HNRNPA2B1, IGF2BP1, IGF2BP2, IGF2BP3, HNRNPC, EIF3A, EIF3B, PRRC2A, ELAVL1, LRPPRC*, and *FMR1*) (12, 13, 25–30). Data were analyzed with the R (version 4.1.1) and R Bioconductor packages.

### m6A Modification and Liver Fibrosis Stage-Related Gene Signature

Distinct m6A modification patterns were identified based on the expression of 28 m6A regulators. A consensus clustering algorithm was used to detect the number of patterns and verify their stability using the ConsensusClusterPlus package in R (31). Differentially expressed genes (DEGs) between the two distinct m6A patterns were determined by the limma package in R (32). The screening criterion was set as adjusted *p* < 0.001. DEGs were correlated against Scheuer score (S) using Spearman's rank correlation to identify m6A-and-stage-related genes and 489 genes were filtered for significance with adjusted *p* < 0.001 and correlation coefficient >0.4 or < -0.4. To quantify the fibrosis stage-related m6A status of individual patients, we defined the 489 m6A-and-stage-related genes as a module and used the moduleEigengenes function in the WGCNA R package to extract the eigengenes (33). The eigengene was the first principal component and represented the expression of all genes in a module termed as m6A-S score. The receiver operating curve (ROC) analysis was used to find the optimal threshold value of the m6A-S score. Patients were classified into significant gene clusters based on the 489 m6A-and-stage related genes using a consensus clustering algorithm for further analysis.

### Gene Ontology Analysis

Gene ontology (GO) analysis was performed based on m6A-and-stage related genes. We used the R clusterProfiler package for GO analysis.

### Gene Set Variation Analysis

To investigate the differentially expressed pathways and biological process activities, Gene set variation analysis (GSVA) that estimates the variation of pathway activity in an unsupervised way was performed using the “GSVA” R package (34). We performed GSVA with the gene sets of “c2.cp.kegg.v7.4.symbols” downloaded from the Molecular Signatures Database (MSigDB). Adjusted *p* below 0.01 and |logFC| > 0.1 were considered as significant.

### Evaluation of Immune Cell Infiltration

Single-sample gene-set enrichment analysis (ssGSEA) was used to quantify the abundance levels of immune cell signatures in each sample in the form of ssGSEA enrichment scores. The gene set including 667 marker genes were based on the study of Charoentong, which contained 23 cell-type-specific sets of both innate immunity and adaptive immunity (Table S3) (35).

### Statistical Analysis

The Wilcoxon-pairs signed rank test, chi-square test, and the Kruskal–Wallis test were used to compare the difference among groups. The value of *p* < 0.05 were considered significant. Correlations of the expression of each m6A regulator, as well as correlations between m6A-S score and immune cells infiltration were visualized by the function “corrplot” in the R package “corrplot.” The principal component analysis (PCA) was used to make data dimensionality reduction and largely retain the information of the original data. The univariable and multivariable ordinal logistic regression analysis was performed to identify risk factors associated with the stage of liver fibrosis. Correlations between the immune cell infiltration and the expression of m6A regulators were computed by Pearson's correlation analyses. Statistical analyses were performed using R version 4.1.1, SPSS23, and GraphPad Prism 9.

## Results

### m6A Regulator-Mediated Modification Patterns Were Related to the Progression of HVB-Related Liver Fibrosis

**Figure 1:**
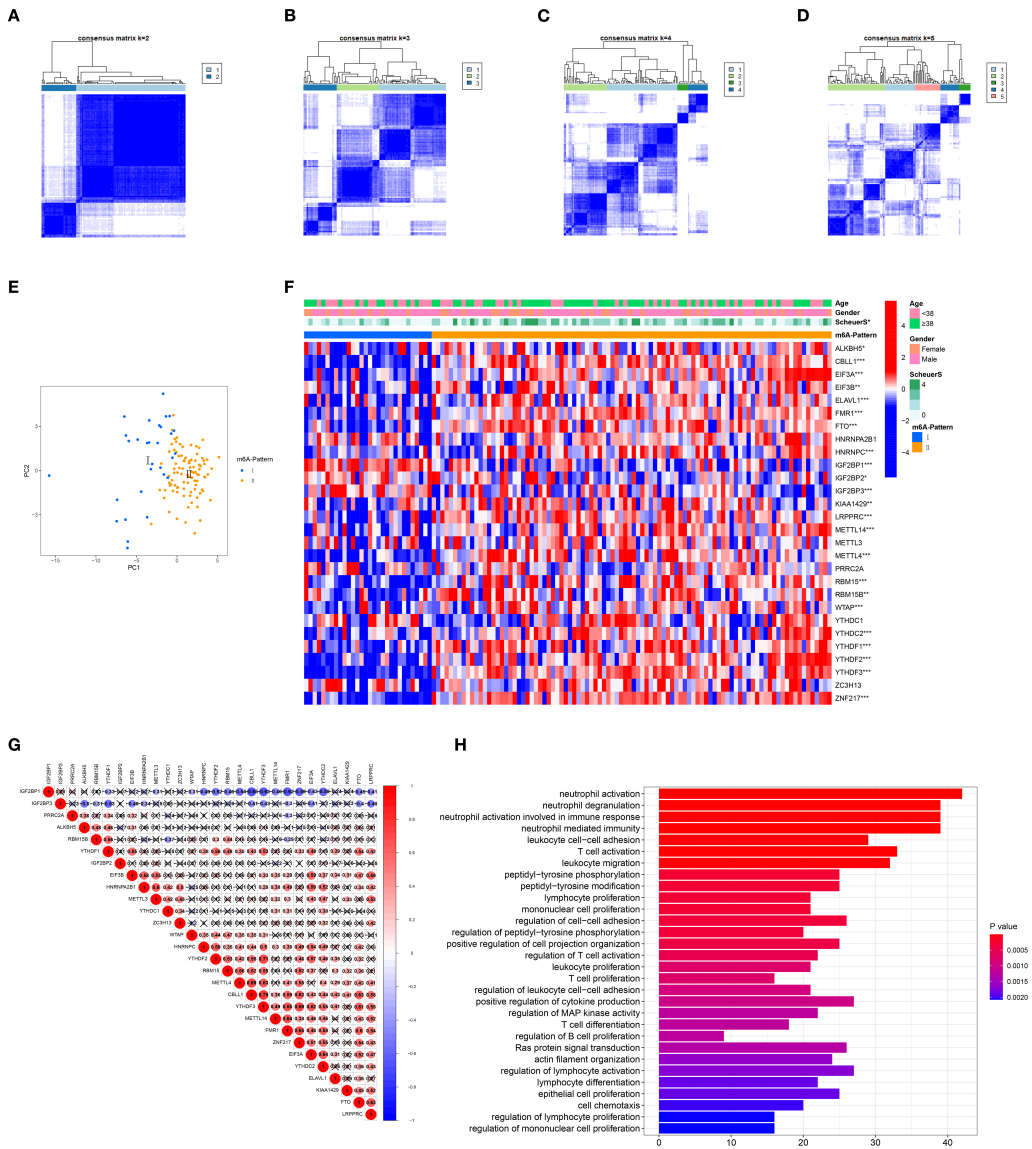
Identification of N6-methyladenosine (m6A) modification patterns. **(A–D)** Consensus clustering of the 28 m6A regulators for *k* = 2–5. **(E)** Principal component analysis (PCA) for the expression profiles of m6A regulators showed a remarkable separation between the two m6A patterns. **(F)** Heatmap of the 28 m6A regulators and clinical traits in m6A-Pattern I and m6A-Pattern II. **(G)** Correlations among the m6A regulators. **(H)** Gene ontology analysis of m6A-and-stage related genes.

The R package ConsensusClusterPlus was used to identify m6A modification patterns based on the expression of 28 m6A regulators. Two distinct modification patterns were classified and termed as m6A-Pattern I and II (Figures 1A–D). According to the PCA results, two m6A patterns could be completely distinguished by the 28 m6A regulators (Figure 1E). There were 30 cases in m6A-Pattern I and 94 in m6A-Pattern II. The stage of liver fibrosis in m6A-Pattern II was significantly more advanced than that in m6A-Pattern I and there were no differences between the two patterns for age and gender (Table 1; Figure 1F). The heatmap was produced to illustrate the differential expression levels of the 28 m6A regulators between the two patterns. *ALKBH5, CBLL1, EIF3A, EIF3B, ELAVL1, FMR1, FTO, HNRNPC, KIAA1429, LRPPRC, METTL14, METTL4, RBM15, RBM15B, WTAP, YTHDC2, YTHDF1, YTHDF2, YTHDF3*, and *ZNF217* showed higher expression in m6A-Pattern II than in m6A-Pattern I, while *IGF2BP1, IGF2BP2*, and *IGF2BP3* showed the opposite. However, *HNRNPA2B1, METTL3, PRRC2A, YTHDC1*, and *ZC3H13* were not differently expressed between the two patterns (Figure 1F). Significant correlations were confirmed among writers, erasers, and readers (Figure 1G).

**Table 1:** Age, gender, and Scheuer score (S) of both N6-methyladenosine (m6A)-Patterns.

	**m6A-Pattern I**	**m6A-Pattern II**	***P-*value**
	***n* = 30 (%)**	***n* = 94 (%)**	
**Age (years)**		
<38	18 (60.0)	38 (40.4)	0.061
≥38	12 (40.0)	56 (59.6)	
**Gender**		
Male	22 (73.3)	66 (70.2)	0.743
Female	8 (26.7)	28 (29.8)	
**Scheuer S**		
0	16 (53.3)	27 (28.7)	0.021
1	7 (23.3)	13 (13.8)	
2	5 (16.7)	28 (29.8)	
3	2 (6.7)	16 (17.0)	
4	0	10 (10.6)	

*The value of p obtained using Pearson's χ^2^-test*.

In total, 6,633 DEGs were selected between the two m6A patterns. Furthermore, a total of 489 genes significantly related to Scheuer score (S) were identified among these DEGs. The GO functional enrichment analysis was applied based on the 489 m6A-and-stage related genes. For the GO biological process (GO BP) analysis, they were in a significant correlation with immunity reactions mediated by neutrophils, T cells, and other leukocytes and they were remarkably related to cytokine production (Figure 1H, Table S2).

### m6A-and-Stage Related Genes Could Separate the Cohort Into Two Gene Clusters With Different Liver Fibrosis Stage and Immune Cell Infiltration

**Figure 2:**
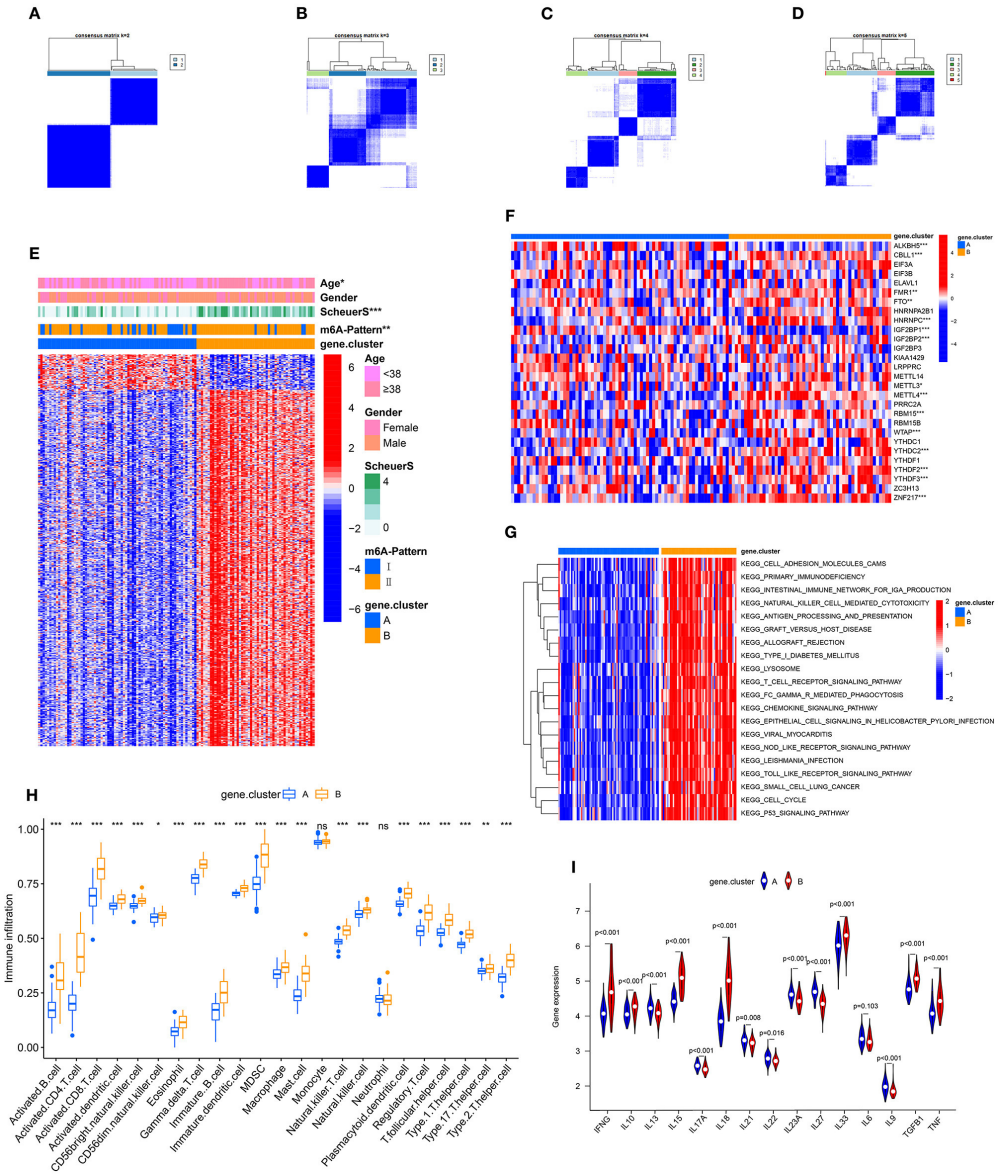
Identification of m6A-and-stage related gene subtypes. **(A–D)** Consensus clustering of the 489 m6A-and-stage related genes for *k* = 2–5. **(E)** Heatmap of the 489 m6A-and-stage related genes and clinical traits in gene clusterA and gene clusterB. **(F)** Expression heatmap of the 28 m6A regulators in gene clusterA and gene clusterB. **(G)** The heatmap of gene set variation analysis (GSVA) enrichment analysis was used to visualize biological pathways between gene clusterA and gene clusterB. **(H)** Different immune cell infiltration of two gene clusters **(I)** Expression of cytokines between the two gene clusters (***p < 0.001; **p < 0.01; *p < 0.05).

To further validate the function of m6A regulation in the progress of hepatic fibrosis, the consensus clustering method was used to divide the chronic HBV infected patients into different genomic subtypes based on the 489 stage-and-m6A related genes. Consistent with the prior m6A modification patterns, two distinct genomic phenotypes were presented, termed as gene clusterA and gene clusterB (Figures 2A–D). There were 71 cases in gene clusterA and 53 in gene clusterB. The heatmap displayed that a large amount of the 489 selected genes were more expressed in gene clusterB than in gene clusterA and the Scheuer score (S) levels were significantly higher in gene clusterB than in gene clusterA (Figure 2E). While the cases in gene clusterB consisted of older ages and more m6A-Pattern II cases than in gene clusterA (Figure 2E; Table 2). We subsequently compared the characters of m6A-Pattern I and II cases in gene cluserA and B. There was no age difference between m6A-Pattern I and m6A-Pattern II cases in gene clusterA (Table 3). However, patients of m6A-Pattern II were older than those of m6A-Pattern I in gene clusterB (Table 3). It was indicated that there was not a correlation of age and m6A modification in patients with mild liver fibrosis, but as the stage progressing it might exist.

**Table 2:** Age, gender, Scheuer score (S), and m6A-Pattern of both gene clusters.

	**Gene clusterA**	**Gene clusterB**	***P-*value**
	***n* = 71 (%)**	***n* = 53 (%)**	
**Age (years)**		
<38	39 (54.9)	17 (32.1)	0.011
≥38	32 (45.1)	36 (67.9)	
**Gender**		
Male	50 (70.4)	38 (71.7)	0.877
Female	21 (29.6)	15 (28.3)	
**Scheuer S**		
0	38 (53.5)	5 (9.4)	<0.001
1	14 (19.7)	6 (11.3)	
2	13 (18.3)	20 (37.7)	
3	5 (7.0)	13 (24.5)	
4	1 (1.4)	9 (17.0)	
**m6A-Pattern**		
I	25 (35.2)	5 (9.4)	0.001
II	46 (64.8)	48 (90.6)	

*The value of p obtained using Pearson's χ^2^-test*.

**Table 3:** Age, gender, and Scheuer score (S) of both m6A-Patterns in gene clusterA and gene clusterB.

	**Gene cluterA**			**Gene clusterB**		
	**m6A-Pattern I**	**m6A-Pattern II**	***P-*value**	**m6A-Pattern I**	**m6A-Pattern II**	***P-*value**
	***n* = 25 (%)**	***n* = 46 (%)**		***n* = 5 (%)**	***n* = 48 (%)**	
**Age (years)**						
<38	14 (56.0)	25 (54.3)	0.894	4 (80.0)	13 (27.1)	0.016
≥38	11 (14.0)	21 (45.7)		1 (20.0)	35 (72.9)	
**Gender**						
Male	19 (76.0)	31 (67.4)	0.448	3 (60.0)	35 (72.9)	0.542
Female	6 (24.0)	15 (32.6)		2 (40.0)	13 (27.1)	
**Scheuer S**						
0	16 (64.0)	22 (47.8)	0.263	0	5 (10.4)	0.668
1	6 (24.0)	8 (17.4)		1 (20.0)	5 (10.4)	
2	3 (24.0)	10 (21.7)		2 (40.0)	18 (37.5)	
3	0	5 (10.9)		2 (40.0)	11 (22.9)	
4	0	1 (2.2)		0	9 (18.8)	

*The value of p obtained using Pearson's χ^2−^test*.

To identify the significant factors that may affect the progress of liver fibrosis stage, an ordinal logistic regression analysis was applied. As shown in Table 4, a univariate analysis showed that age, m6A-Pattern, and gene cluster were significant factors for predicting the progress of liver fibrosis stage with *p* < 0.05 and they were selected into the multivariate ordinal logistic regression model. Increasing odds of the progress of disease stage was associated with age ≥38 years [odds ratio (*OR*) = 3.155, 95% *CI*: 1.561–6.375, *p* = 0.001] and gene clusterB (*OR* = 8.209, 95% *CI*: 3.773–17.862, *p* < 0.001), which could be considered as independent predictors.

**Table 4:** Ordinal logistic regression result.

**Variables**	**Univariate analysis**			**Multivariate analysis**		
	**OR**	**95% CI**	***P-*value**	**OR**	**95% CI**	***P-*value**
**Age (years)**						
≥38	3.640	1.860–7.121	<0.001	3.155	1.561–6.375	0.001
<38	1.000			1.000		
**Gender**						
Male	0.922	0.460–1.848	0.820			
Female	1.000					
**m6A-Pattern**						
II	3.518	1.594–7.776	0.002	1.760	0.756–4.093	0.190
I	1.000			1.000		
**Gene cluster**						
B	9.810	4.632–20.775	<0.001	8.209	3.773–17.862	<0.001
A	1.000					

*OR, odds ratio; CI, confidence interval*.

Between the two gene clusters, there were significantly different expressions of *ALKBH5, CBLL1, FMR1, HNRNPC, IGF2BP1, IGF2BP2, METTL3, METTL4, RBM15, WTAP, YTHDC2, YTHDF2, YTHDF3*, and *ZNF217* as shown in Figure 2F. GSVA enrichment analysis was performed to explore the pathways that are differentially expressed between two gene clusters. Gene clusterB presented enrichment pathways associated with immune, stromal, and cacinogenic activation, such as the activation of chemokine signaling pathway, toll-like receptor signaling pathway, T cell receptor signaling pathway, and p53 signaling pathway. While gene clusterA was shown relatively less immune active (Figure 2G, Table S1). The results of the ssGSEA analysis showed evidently more immune cell infiltration in gene clusterB than in gene clusterA (Figure 2H, Table S4), which suggested that m6A modification might interact with infiltrating immune cells in the progress of liver fibrosis. We then compared the cytokines distribution across the two gene clusters. *IFNG, IL10, IL15, IL18, IL33, TGFB*, and *TNF* were observed significantly high expression in gene clusterB, while *IL13, IL17A, IL21, IL22, IL23A, IL27*, and *IL9* have shown dramatic high expression in gene clusterA. *IL6* showed no clear distinction between the two groups (Figure 2I).

### Individual m6A Modification Was Quantified and Was Associated With Immune Cell Infiltration and the Progression of Liver Fibrosis

**Figure 3:**
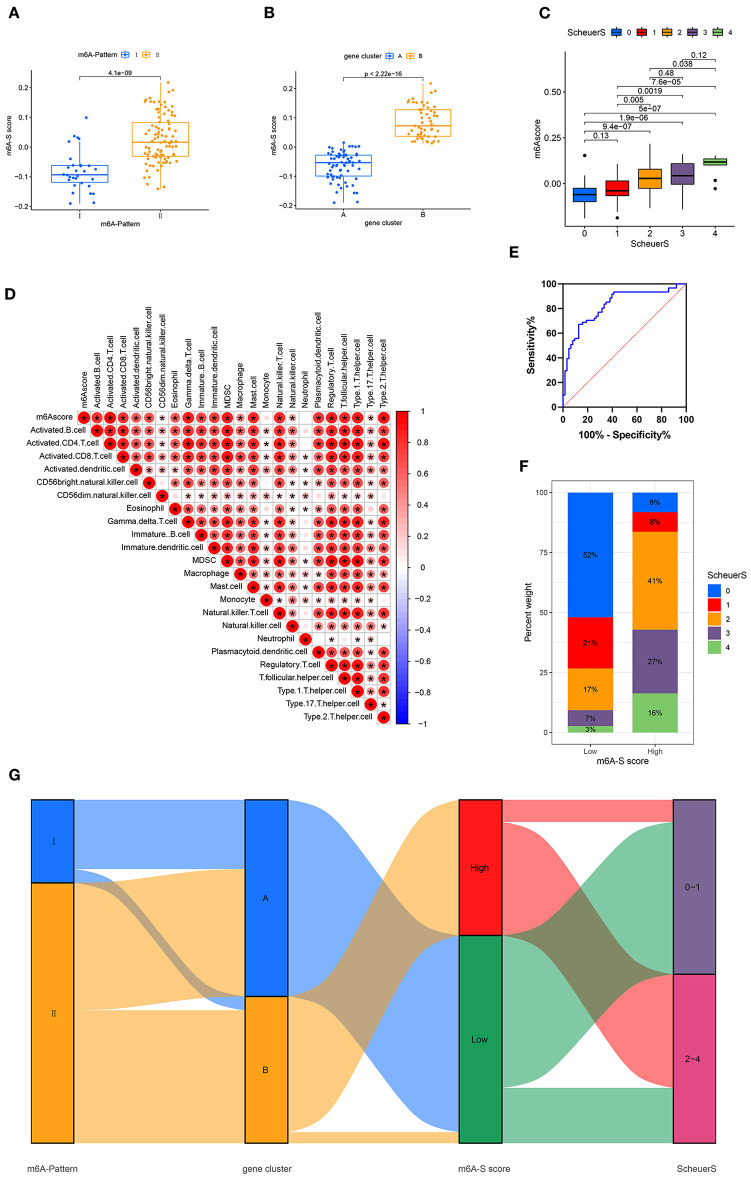
Construction of m6A-S score. **(A)** Difference of m6A-S score between two m6A modification patterns. **(B)** Difference of m6A-S score between two gene clusters. **(C)** Comparison of m6A-S scores among patients in different liver fibrosis stages. **(D)** Correlations between m6A-S score and infiltrating immune cells. **(E)** The receiving-operating characteristic (ROC) curve analysis of the m6A-S score were used to differentiate the mild and severe stage of liver fibrosis. The area under the curve was 0.820 with *p* < 0.001 and the 95% *CI* was 0.754–0.902. **(F)** The proportion of disease stages in high and low m6A-S score group. **(G)** Sankey diagram showing the relationship among m6A patterns, gene clusters, m6A-S score groups, and fibrosis severity.

Based on these m6A-and-stage related genes, we utilized the moduleEigengenes function in the WGCNA R package to construct a set of scoring system, termed as the m6A-S score for quantifying m6A modification in the process of liver cirrhosis (Table S5). The m6A-S scores were obviously higher in the m6A-Pattern II than in the m6A-Pattern I (Figure 3A) and higher in gene clusterB than in gene clusterA (Figure 3B). As shown, the m6A-S score increased as the stage progressed (Figure 3C). It demonstrated a positive correlation between m6A-S score and immune cell infiltration in patients with CHB (Figure 3D).

Then, 49 patients were selected in the high m6A-S score group and 75 in the low, based on the optimal cut-off value 0.019 defined by an ROC curve. The area under the ROC curve was 0.820 with *p* < 0.001 and the 95% *CI* was 0.754–0.902 (Figure 3E). The stage of liver fibrosis in the high m6A-S score group was more progressed than the low m6A-S score group (Figure 3F). The relationships among m6A-Patterns, gene clusters, m6A-S score groups, and Scheuer score (S) were visualized in a Sankey diagram (Figure 3G).

### *ALKBH5* Interacting With the Macrophage and *WTAP* Interacting With Nature Killer T Cell Were Key Genes and Immune Cells in HVB-Related Liver Fibrosis

**Figure 4:**
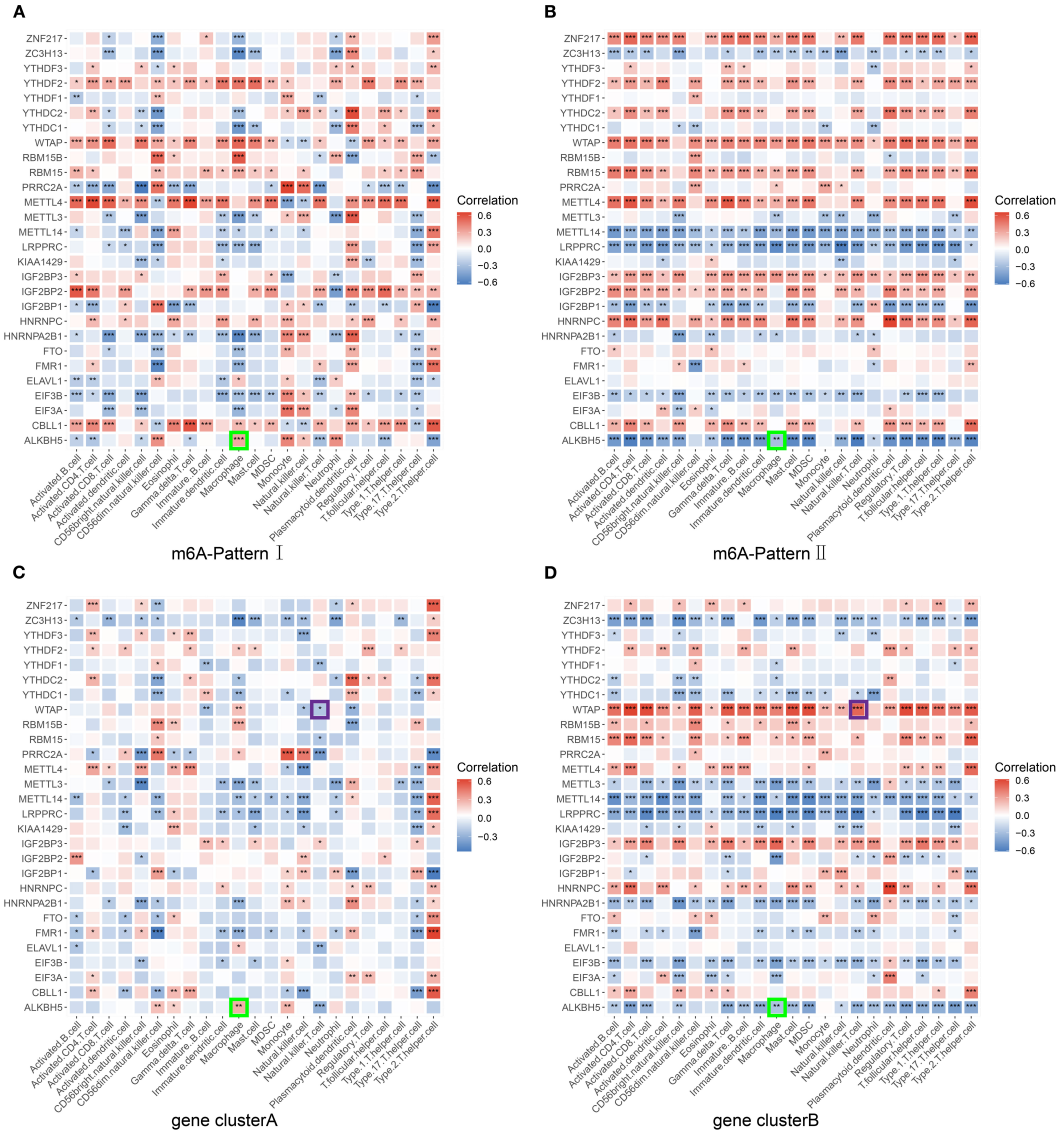
Screening for critical genes and immune cells. The correlation between each infiltrating immune cell type and each m6A regulator was analyzed using Pearson's correlation analyses and compared in different pairs of subgroups. **(A)** Patients of m6A-Pattern I. **(B)** Patients of m6A-Pattern II. **(C)** Patients of gene clusterA. **(D)** Patients of gene clusterB (***p < 0.001; **p < 0.01; *p < 0.05).

We have set up three different grouping conditions: m6A-Pattern, gene cluster, and m6A-S score group. Additionally, in the group of m6A-Pattern I or II, patients might have different characters with high m6A-S scores compared with low m6A-S scores, which was the same in gene clusterA or B. We correlated the expression of m6A regulators against the infiltrating immune cells in each group mentioned above and chose the significantly opposite correlation coefficients as significant results between each pair of groups. As there were not large enough sample sizes, patients with high or low m6A-S scores were not compared in the gene clusterA and gene clusterB. Critical genes and immune cells were selected when they showed significant results in at least 4 pairs of groups. Then, we took the intersection of these selected genes and the differentially expressed m6A regulators in m6A-Patterns and gene clusters to identify the most relevant genes. Finally, *ALKBH5* interacting with macrophage and *WTAP* interacting with natural killer T cell were considered key points of m6A modification regulation in the progression of liver fibrosis (Figure 4; Supplementary Figure 1).

## Discussion

Chronic hepatitis B is a dominant etiology of liver cirrhosis. Although the mechanisms of the disease progression have been extensively studied in the past, the role of m6A modification in the process remains unclear. In this study, 2 distinct m6A modification patterns were distinguished based on the 28 m6A regulators. The DEGs between two m6A-Patterns were screened out and then filtered by the correlation with Scheuer score (S). Four hundred and eighty-nine obtained genes were considered m6A-and-stage related signature genes, based on which two genomic subtypes were identified. With the more advanced disease stage, gene clusterB was enriched in immune active signaling pathways and filled with more immune cells. Cytokines expressed differently between the two gene clusters. For quantifying the individual heterogeneity of m6A modification, the m6A-S score was constructed based on these m6A-and-stage related genes. The m6A-S score was significantly related to the immune cell infiltration and increased with the increasing fibrosis stage. Finally, critical genes and immune cells were selected which might take part in m6A modifications influencing the progression of liver cirrhosis.

It is reported that m6A modification plays an important role in HBV infection and virus life cycle. According to Siddiqui's studies, it was demonstrated that m6A modification could regulate the life cycle of HBV (22, 36, 37). They reported that m6A modification of RNA took part in the IFN-α induced HBV RNA degradation (38). It could be reasonably speculated that there would be a difference of m6A modification between HBV infected patients and healthy control, which had not been proven in our study due to the lack of proper cases.

No difference of age between the two distinct m6A patterns was found. As we narrowed the grouping criterion more precisely to the stage of liver cirrhosis, the patients in gene clusterB showed older with more advanced liver cirrhosis stage than in the gene clusterA. There was no particular age difference between patients with different m6A-Patterns in gene clusterA, while in gene clusterB patients with m6A-Pattern II were significantly older than patients with m6A-Pattern I. It could be concluded that m6A modification might interact with the liver fibrosis regardless of its stage. However, age did not seem to affect the liver fibrosis when it was mild, while age could interact with fibrosis as it progressed severely. In view of the chronic course of liver cirrhosis, our inferences were reasonable.

The key immune cells and m6A regulators might play an important role in the progress of liver fibrosis. Kwon and Choi (39) demonstrated that the depressed toll-like receptor signaling pathway may reduce pro-inflammatory cytokines and macrophage accumulation preventing the hepatic fibrosis. Similar results could be seen in our study. In contrast to gene clusterB, gene clusterA exhibited a depression of toll-like receptor signaling with significantly less macrophage infiltrating. Besides, in gene clusterB, *ALKBH5* expression levels were lower than in gene clusterA and its expression inversely associated with the macrophage infiltration suggesting it as a protective factor of liver cirrhosis (Figure 4D).

Jin's study showed similarly that the number of hepatic NKT cells increased as the HBV-tansgenic mice aging and treated with hepatotoxin carbon tetrachloride. They further confirmed that NKT cells were critical for hepatic stellate cells activation (40). In our study, gene clusterB with severer liver fibrosis showed more infiltrating NKT cells and more aged patients. *WTAP* was more expressed in gene clusterB than in gene clusterA and positively associated to the NKT cell infiltration in gene clusterB and in overall trend (Figure 4D; Supplementary Figure 1G). *WTAP* was reported to improve the HCC progression (41, 42). Taken together, these findings suggest a role for *WTAP* interacting with NKT cells in the progress of HBV-related liver fibrosis.

In the progress of HBV-related liver fibrosis, cytokines are significantly involved in immune regulation and inflammation. They not only enhance the virus clearance, but also participate in liver damage. Prior studies have shown that IL10 inhibits cytokine production, regulates T cell immunity, thus facilitates immune tolerance, and persists HBV infection advancing hepatic fibrosis (43, 44). It is controversial for the effect of IL21, which can regulate both cellular and humoral immune responses (45). IL21 was reported to play an important role in the viral clearance as a therapeutic agent (46). On the other hand, it was closely related to the progression of liver cirrhosis and liver injury (44, 45). The immune response is a complicated dynamic network mediated by various immune cells and cytokines in the progress of HBV-related liver fibrosis (2, 44, 47). Our study illustrated a distinct cytokine discrepancy between two gene clusters, suggesting a significantly different immune response in diverse m6A modification status during the progress of HBV-induced liver fibrosis. However, the specific mechanism remains to be investigated in-depth in future studies.

In view of the need for quantifying individual m6A modification heterogeneity, we established a set of scoring systems based on the 489 m6A and liver fibrosis staging-related genes. The genes were defined as a module and the module eigengene in WGCNA was extracted as the m6A-S score, which was a representative of the gene expression profile. In our study, the m6A-S score increased as fibrosis progressed and positively related to numerous immune cell infiltration. Besides, m6A-Pattern II and gene clusterB separately exhibited higher m6A-S scores than m6A-Pattern I and gene clusterA. These results further suggested the idea that the m6A-S score was a potent and stable measure for comprehensive evaluation of individual m6A modification patterns in patients with CHB and closely correlated to the progression of liver cirrhosis.

In conclusion, m6A modification is involved in the progress of HBV-related liver fibrosis and it may interact with immune effects, which provides novel ideas to the prevention and diagnosis of liver cirrhosis and HCC.

## Data Availability Statement

Publicly available datasets were analyzed in this study. This data can be found here: https://www.ncbi.nlm.nih.gov/geo/query/acc.cgi?acc=GSE84044.

## Author Contributions

TZ, HW, and QZ were responsible for the study concept and design. TZ, HW, and JQ performed statistical analysis and contributed to the writing of the manuscript. TZ and TL performed literature search and data collection. TL and QZ improved the language. All authors have contributed significantly and approved the final version of the manuscript.

## Funding

This work was supported in part by grants from the National Natural Science Foundation of China (82160124, 82100641 and 82103075), the Clinical Medical Science and Technology Innovation Program (202019094), and the WBE Liver Fibrosis Foundation (CFHPC2021011).

## Conflict of Interest

The authors declare that the research was conducted in the absence of any commercial or financial relationships that could be construed as a potential conflict of interest.

## Publisher's Note

All claims expressed in this article are solely those of the authors and do not necessarily represent those of their affiliated organizations, or those of the publisher, the editors and the reviewers. Any product that may be evaluated in this article, or claim that may be made by its manufacturer, is not guaranteed or endorsed by the publisher.
