# Supplementary figures and images for: N6-Methyladenosine Modification Participates in the Progression of Hepatitis B Virus-Related Liver Fibrosis by Regulating Immune Cell Infiltration
Source: Front Med (Lausanne). 2022 Mar 2;9:821710. doi: 10.3389/fmed.2022.821710 (PMC8924664; doi:10.3389/fmed.2022.821710)

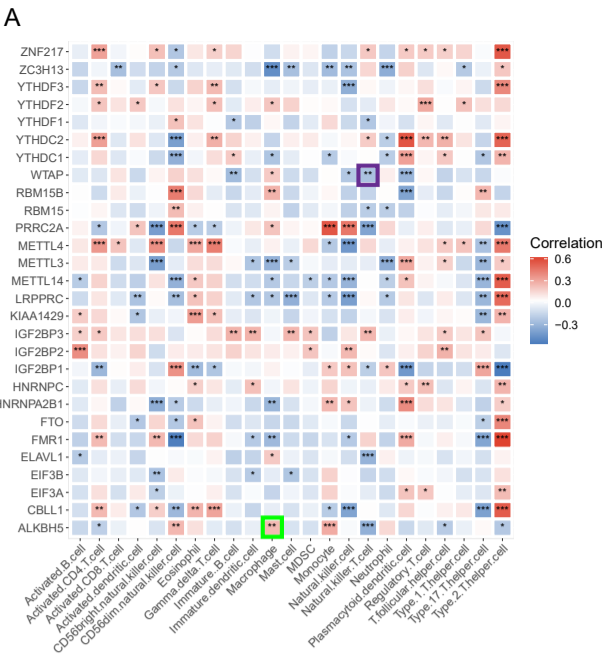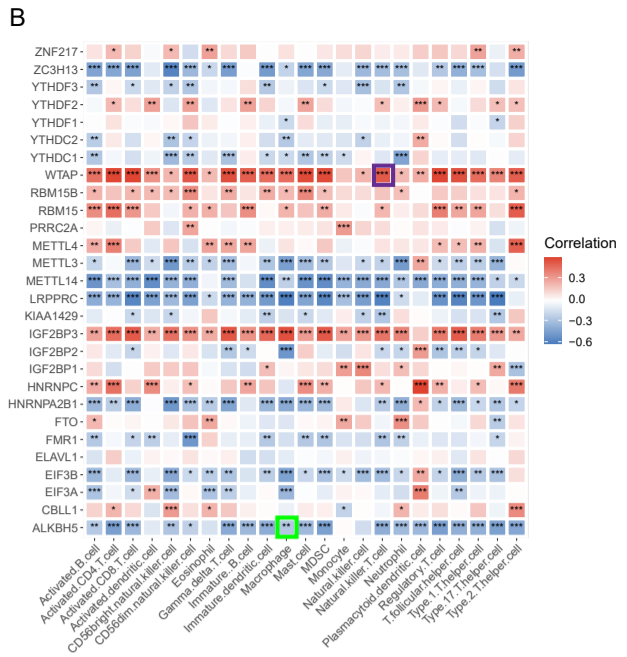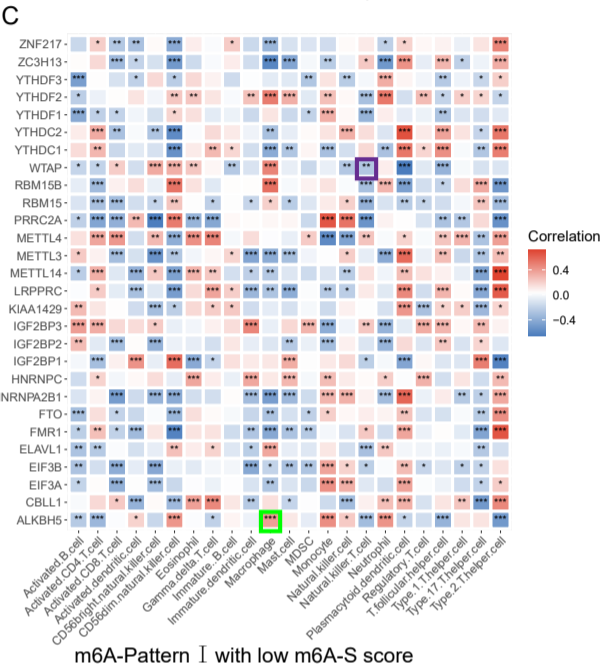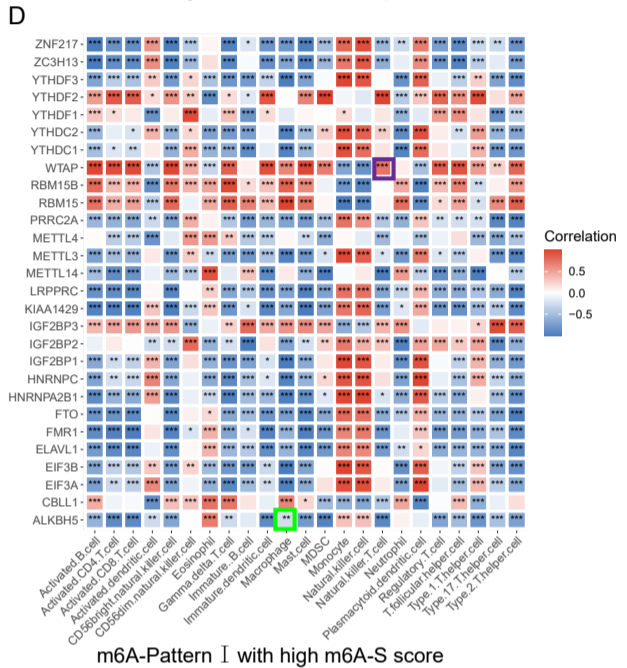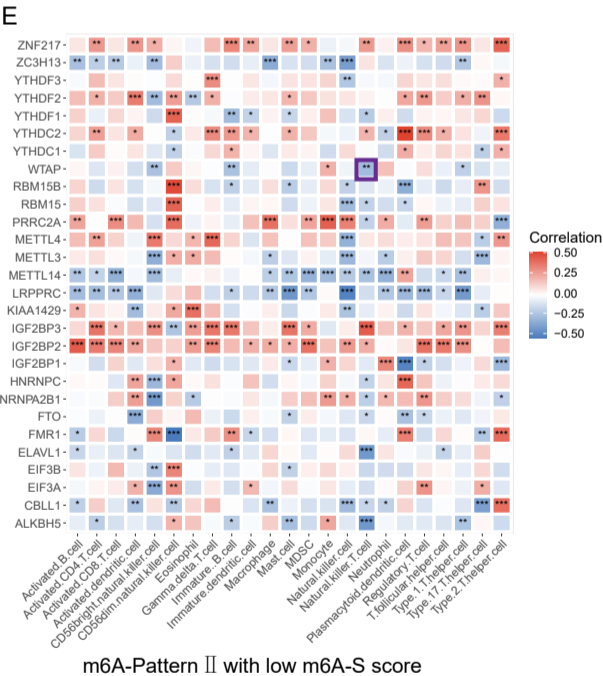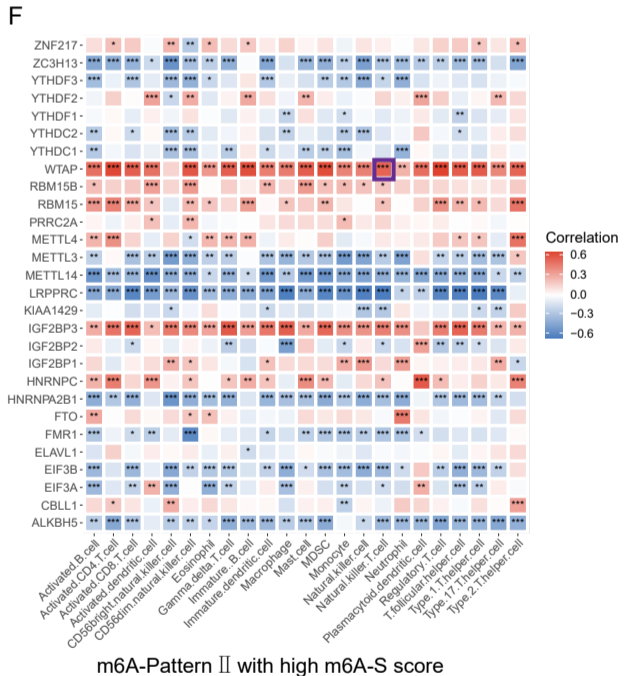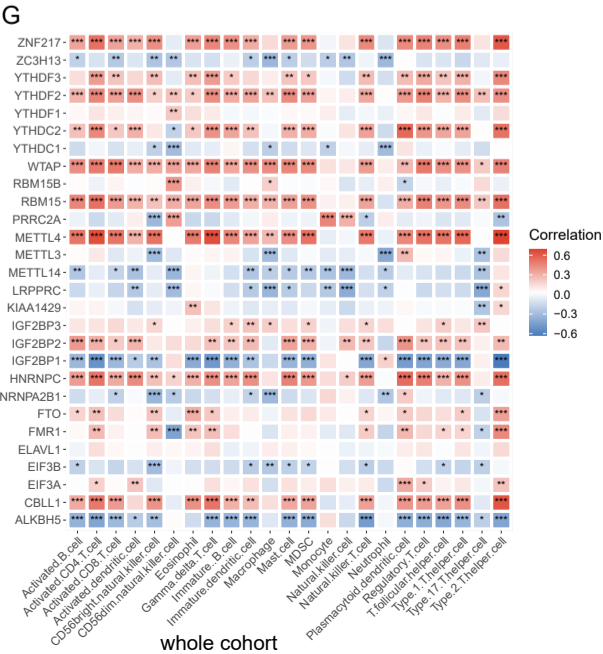

Supplement: Supplementary file 1 [file Data_Sheet_2.pdf]
