# Supplementary material for: N6-Methyladenosine Modification Participates in the Progression of Hepatitis B Virus-Related Liver Fibrosis by Regulating Immune Cell Infiltration
Source: Front Med (Lausanne). 2022 Mar 2;9:821710. doi: 10.3389/fmed.2022.821710 (PMC8924664; doi:10.3389/fmed.2022.821710)
Supplement: Supplementary file 3 [file Data_Sheet_1.docx]

Supplementary Material

# Supplementary Figures and Tables

## Supplementary Figures

**Supplementary Figure 1.** The correlation between each infiltrating immune cell type and each m6A regulator were analyzed using Pearson correlation analyses and compared in different pairs of subgroups. **(A)** patients of low m6A-S score group **(B)** patients of high m6A-S score group **(C)** patients of m6A-Pattern Ⅰ with low m6A-S score **(D)** patients of m6A-Pattern Ⅰ with high m6A-S score **(E)** patients of m6A-Pattern Ⅱ with low m6A-S score **(F)** patients of m6A-Pattern Ⅱ with high m6A-S score **(G)**patients of the whole cohort (***P < 0.001; **P < 0.01; *P < 0.05)

## Supplementary Tables

**Supplementary Table 1.** The activation states of biological pathways in two gene clusters by GSVA enrichment analysis

**Supplementary Table 2.** GO analysis for 489 m6A-and-stage related genes

**Supplementary Table 3.** The gene sets used for marking each infiltrating cell type

**Supplementary Table 4.** The results of estimating relative abundance of infiltrating immune cells in 124 chronic HBV infected patients

**Supplementary Table 5.** Clustering, m6A-S score Scheuer score(s) and age of the cohort
